# What is the role of scientists in meeting the environmental challenges of the twenty-first century?

**DOI:** 10.1098/rsos.240498

**Published:** 2024-06-19

**Authors:** Richard D. Gregory, Jon Bridle, Jeremy D. Wilson

**Affiliations:** ^1^ RSPB Centre for Conservation Science, Sandy, Bedfordshire, UK; ^2^ Centre for Biodiversity & Environment Research, Department of Genetics, Evolution and Environment, University College London, London, UK; ^3^ RSPB Centre for Conservation Science, 2 Lochside View, Edinburgh Park, Edinburgh EH12 9DH, UK

**Keywords:** biodiversity loss, climate change, science, policy, activism, conservation

## Abstract

We live at a time of rapid and accelerating biodiversity loss and climate change that pose an existential risk to the environment, humanity, and social justice and stability. Governmental responses are seen by many citizens, including scientists, as inadequate, leading to an increase in civil protests and activism by those calling for urgent action to effect change. Here we consider the role(s) of scientists in responding to those challenges and engaging with policy given that when a scientist moves into political advocacy, reflecting their values and preferences, their objectivity and the value of scientific opinion may be seen as compromised. We then consider whether institutional setting and career stage may affect decisions to engage with policy or activism. Against this backcloth, we ask whether it is sufficient for scientists to act as impartial ‘brokers’ in societal decisions, arguing they should consider acting as ‘*Honest Advocates*’ in policy formation in some circumstances. Such advocacy can contribute to decision-making in a purposeful, well-informed manner, doing societal good without damaging the reputation of science. We encourage scientists to each reflect on their multiple roles in addressing the environmental challenges of our time.

## 1. Introduction

At a time of accelerating and potentially dangerous levels of environmental change, there are increasing calls for scientists to be more proactive in political debate and policy making, and in civil disobedience, beyond their more traditional role of informing evidence-led policy [1–3]. There is however no consensus on when and where such direct intervention is appropriate. Some argue that scientists have a moral duty as citizens to stand up and be counted, arguing that they cannot continue to influence policy from the margins when human civilizations are facing an existential threat [2]. Others argue this is a step too far and it is not a scientist's role to address normative questions in society, namely those involving value judgements [4]. In this article, we revisit this debate and explore the role of science and scientists during a time of severe and arguably existential environmental challenges.

Science is defined as the systematic study of the world by observation and experiment to apply knowledge based on the explanations for these data that contain fewest assumptions. The scientific method is characterized by accuracy, precision, repeatability and impartiality, and uses communal mechanisms such as critiques, peer review, open discussion, conferences and independent assessment to scrutinize, validate and update scientific claims [5,6]. Scientific advances have a pervasive influence on human lives and livelihoods, and science continues to be a dominant philosophy in building knowledge, advancing technology, making predictions in time and space, and improving the quality of human life.

Some of the most pressing challenges of the twenty-first century concern the interlinked global crises of accelerating biodiversity loss and climate change [7] (box 1). These pose an existential risk to human life, prosperity, peace and sustainability, because they have cascading and almost exclusively negative effects on human health and wellbeing, infectious disease risk, social inequalities, human migration, food production and climate regulation [7,8]. For example, three of the top four long-term global risks identified in the World Economic Forum's 2024 report were related to biodiversity and ecosystems [10]. The evidence base for each is rich and compelling, with remarkably high levels of scientific consensus in its interpretation [9,11]. In addition, the direct evidence of the consequences of climate change, or at least its extremes, is increasingly visible. Heat waves, droughts, wildfires, storms and flooding now make headline news across the world, and their unprecedented frequency make them a political and economic reality from local to global scales. By contrast, however, although some environmental loss is evident and attracts headlines, such as forest clearance, coral bleaching and threats to iconic species, the long-term loss of biodiversity is less tangible because it is technically difficult to quantify, is statistically noisy and is often at its most severe in places remote from centres of political or economic influence. Moreover, we suffer from ‘shifting baselines’ so that our perception of change, even within generations, is dulled [12]. Nonetheless, the loss of nature is severe, continuing and global, and often experienced most severely by the world's least economically and politically powerful [13].

Box 1. **Arguably some of the most pressing societal challenges of the twenty-first century relate the interlinked global crises of accelerating biodiversity loss and climate change.**

**Accelerating biodiversity loss.** The latest report by the Intergovernmental Science-Policy Platform on Biodiversity and Ecosystem Services [8] concluded (1) that nature and its vital contributions to people in the form of biodiversity and ecosystem functions and services were deteriorating worldwide, (2) that direct and indirect drivers of change have accelerated during the past 50 years, and (3) that goals for conserving and sustainably using nature would not be met on current trajectories and would only be met through transformative changes across economic, social, political and technological systems.

**Accelerating climate change.** The latest report of the Intergovernmental Panel on Climate Change [9] concluded that (1) human activities, principally through emissions of greenhouse gases, have caused global warming with global surface temperatures rising, (2) greenhouse gas emissions have continued to increase driven by unsustainable energy use, land use and land-use change, lifestyles and patterns of consumption and production, (3) climate change was affecting many weather patterns and climate extremes, leading to widespread adverse impacts on nature and people, and (4) vulnerable communities around the globe who have contributed the least to current climate change historically, are disproportionately affected now. Looking forwards, the IPCC predicts that continued greenhouse gas emissions will increase global warming and each increment of warming is predicted to intensify multiple and concurrent risks to humanity.

Despite the severity of climate and biodiversity trends, the commitments made by governments and the targets set by the parties to the Convention on Biological Diversity (CBD) in 2002 and 2010 have very largely not been met [14–16]. A further set of progressive action targets for 2030 was adopted by the CBD in December 2022 within the Kunming-Montreal Global Biodiversity Framework [17]. Time will tell whether these laudable ambitions are realized. Similarly, on climate change, mitigation targets to reduce greenhouse gas emissions and related climate-related actions from the Conference of the Parties of the UNFCCC have come and gone, and successive scientific assessments show that agreed goals have not been met [9]. There is a societal risk that repeated failures will discourage further progress, by making climate mitigation targets seem increasingly unrealistic. Perversely, the result of successive failure to meet targets may be that targets are weakened rather than strengthened through time.

Most scientists and learned bodies judge the speed and scale of action to address the climate and nature crises as insufficient (e.g. [9,11]). However, decisive action is effectively delayed by vested interests who may demand greater scientific certainty, pit scientists against each other, and perpetuate illusions of scientific doubt [18]; or adopt plain blocking tactics. The slow response has led to public frustration and anger and to the rise of activism from younger generations, as well as among many scientists, ranging from the passive and indirect to the active and direct activism (box 2). Young activists such as Greta Thunberg have become global media stars and the ‘School Strike for Climate’ was a global phenomenon attracting intense media interest and putting a spotlight on the severity and implications of global warming. It has put increasing pressure on industry and politicians responsible for addressing climate change and demanded that governments honour their political commitments, often promised in their election manifestos. State and non-state actors from the Global South are increasingly visible and adding their voices to intergovernmental debate on the environment too. Grassroots activism can be self-organized or coordinated, seeking to build a moral and emotive case to justify sometimes disruptive actions. Protests are typically non-violent, often designed to attract media interest, but in some jurisdictions may be judged illegal, warranting arrest and punitive action. The emergence of decentralized, international and politically neutral movement groups such as *Extinction Rebellion* [19] reflects this general trend and mood in society. These groups aim to use non-violent action and civil disobedience to persuade governments to act urgently on the climate and ecological crises.

Box 2. **Activism exists as a spectrum of activity from passive and indirect through to active and direct action under a number of different banners and terms.**

**Table:** 

Passive/indirect	→	Active/direct
Letter writing, electoral activity, purchasing power, petition signing, flyposting, displaying banners, lifestyle choices	Public speaking, spokesperson, social commentary, petition promotion, diplomacy, negotiations, campaigning, local practical actions (e.g. litter picking, tree planting)	Non-violent direct action, civil disobedience/resistance, performance art, trespass, sit-ins, strikes, marches, boycotts, workplace and other occupations, road blocking/obstructing, door-stepping

Against this background, some scientists at different career stages have chosen to move visibly towards activism in its different forms and to different degrees in their professional capacities. For example, *Scientists for Extinction Rebellion* is led by several well-established UK scientists, taking action to confront what they see as an impending ecological breakdown [20] and *Scientist Rebellion* is a similar international coalition [21]. A recent open letter from *Extinction Rebellion* addressed to COP28, and signed by over a thousand scientists, concludes ‘As scientists and academics, we believe it is now necessary to step up and engage in collective climate action’. The language is strong, blunt and direct. Other scientists may not engage with such activism in a professional capacity, though some do in their personal lives. Many scientists now face a dilemma in how best to balance a successful scientific career alongside their values and desire to act at a time of unprecedented environmental change and perceived risk. Many are also challenged directly about their responsibility to take direct protest action, given their expertise and knowledge, by their students, their peers, family and friends; the case being made that scientists' involvement brings gravitas to such protests and causes. The failure of scientists to be involved in direct action might convince others that they do not think the situation is as serious as some argue.

Here we explore some of the challenges, opportunities and choices that face scientists in their professional lives at the interface of science, policy and activism. First, we consider the established role of scientists in society and four conceptualized roles of a scientist in public policy making. We then focus on the role of scientists in biodiversity conservation, building on long-running debate in the field of conservation biology. Next, we consider the impact of the institutional setting on science and scientists, comparing academia with environmental non-governmental organizations (eNGOs), using the Royal Society for the Protection of Birds (RSPB) as a case study. Next, we look at the challenges and risks at the coalface of science and policy. Finally, we ask whether it is enough for scientists to be detached ‘honest brokers’ in policy formation and suggest a way forward.

## 2. The discipline of science for conservation: the role of mission and values

### 2.1. What is the role of a scientist in society?

The scientific assessment of policy options assists societal decision making. Science and scientists have many stakeholders to consider and inform, spanning governments, policy makers and advisers, businesses, educational institutes, fellow researchers and wider society. Within this complex, views on the purpose of science and its value vary by sector and by issue, making the science–policy interface a challenging environment. Good governance uses the best quality scientific information and interpretation to make legislative and policy decisions on major societal issues, such as health, food and energy production, security and the environment, in the public interest. It is the role of scientists to help governments and policy makers involved in such complex decisions to understand the scientific basis for the choices on offer, the assumptions being made and their tolerances and uncertainties, and the options available to them, so that the decisions are well-informed while recognizing that other factors beyond the scientists' remit will also play a part. To be effective in this role a scientist must understand the perspectives, roles and needs of the policy and decision makers and be able to communicate their findings and conclusions openly and comprehensibly. This works best when those receiving the information are also scientifically literate in the subject area.

But should scientists simply inform decision-making or participate in it? Pielke [22] proposed four idealized expert roles for a scientist in policy making. This simplified model highlights potential differences in roles, purposes and motivations that four different scientist archetypes may have. A scientist might naturally fit into one of these roles or would be more likely to move between them depending on context. The motivation of the ‘*Pure Scientist*’ (role 1) is to pursue research out of intrinsic interest and curiosity without immediate ‘applied’ interest. They do not wish to interact with policymakers beyond indirectly through the publication of peer-reviewed science and its dissemination through formal channels. A ‘*Pure Scientist*’ does not see it as their professional role to enter the world of policy and politics and might be ill-equipped to do so given their career path, experience, and skill set.

Pielke's other three roles engage with policy and decision makers but to different degrees and with different purposes. The ‘*Science Arbiter*’ (role 2) seeks to engage and answer factual questions and queries posed by policymakers to remove any ambiguity around the science and evidence, and therefore *indirectly* inform decision making, while removing their own agency from the discussion. The ‘*Issue Advocate*’ (role 3) comes armed from their own analysis and value set with a preferred view and focuses on the implementation of that specific policy option, rather than offering a range of different options. They act to limit choices. Finally, the ‘*Honest Broker of Policy Alternatives*' (role 4) aims to provide an impartial overview of all scientifically legitimate policy alternatives, strengths and weaknesses and their tolerances and uncertainties, so broadening the scope of rational policy options and their outcomes. They act for informed choice by the decision maker and thereby aim to improve decision making [23,24]. Pielke also observes that ‘*Pure Scientist*s’ and ‘*Science Arbiters*’ can, inadvertently or intentionally, slip into ‘stealth mode’ and act as advocates should they begin to present information that is partial or biased. Pielke's heuristic model illustrates that scientists often have a choice about the role they choose to play in political debates and policy formation, but this is not a value-free space in any discipline [6], and certainly not in mission-driven science such as that which informs conservation.

### 2.2. The role of values in the mission-driven science of conservation

The field of conservation biology emerged in the 1980s. Like other fields informed by science, such as public health and education, it is mission-driven by name. Conservation biology has openly wrestled with its purpose, objectivity, and whether conservation scientists should be advocates in public policy debates [25–27]. Even though conservation biologists have been described as ‘physicians to nature’ (e.g. [28]), the persistence of disciplinary self-doubt in the science of conservation contrasts with medical science, where the mission to do good sits easily alongside the highest standards of scientific practice and evidence-based decision making. Why is there little sense of moral panic in medicine when its experts share clear policy goals concerning public health and behaviour change, whereas concerns abound among the media and politicians when conservation scientists share similar goals concerning planetary health?

In contrast to medical scientists, conservation biologists have been accused of ‘overstepping [their] role as scientists' and ‘stepping out of a scientific role and into the role of a policy advocate’ [29]. Such accusations led many to argue that conservation biologists needed to acknowledge and communicate their own values, and the value-driven goals that underlie their research and policy recommendations more clearly [26,30]. Ambitions to protect endangered tigers, species richness, genetic diversity, pristine wilderness or functioning ecosystems are all legitimate, but are different, raising the question of the goal of conservation, and whom it serves [31]. However, to recognize that conservation biology is a value-driven and goal-orientated science does not discredit its value to society in rational decision making (as medical science illustrates), and no science can completely avoid value judgements associated with interpretation of information, nor the social setting in which policy discussion plays out [30].

To be credible, any science needs to be transparent in its reasoning, methods and recommendations, and open to criticism, revision and correction, but this does not require its operatives to be devoid of moral values or opinions, provided their data and interpretation are open to scrutiny [6]. The risk in not doing so is that public trust in science will be undermined, which would be counterproductive to achieving conservation or wider environmental goals [24,32]. That said, there remain many barriers that limit the translation of science into conservation policy, including a current lack of political priority, powerful vested interests, poor engagement between scientists and decision-makers, and the fact that conservation problems and their solutions are often complex and uncertain, given the multi-dimensional and intricate nature of biodiversity itself [33].

Applying Pielke's model in the conservation context, many biodiversity and environmental scientists and key scientific roles in society could be best described as a ‘*Science Arbiter*’ or ‘*Honest Broker*’ at least in their professional capacities and in their areas of expertise, often working cooperatively with others to appraise environmental information and provide authoritative descriptions of past and current patterns and trends, and to derive conclusions that inform decision making. Many such scientific assessments have proven influential globally in policy framing and formation. For example, the influential ‘Stern review’ [34] assessed evidence on the impacts of climate change on economic costs. The Millennium Ecosystem Assessment [35] assessed the consequences of ecosystem change for human well-being, involving consensus-formation by over a thousand experts worldwide. This was followed by the Intergovernmental Science-Policy Platform on Biodiversity and Ecosystem Services (IPBES)'s Global Assessment of Biodiversity and Ecosystem Services [36], which was led by around 150 experts, plus 350 contributing authors from all around the globe. IPBES was set up in 2012 as an independent intergovernmental body to strengthen the science–policy interface for the conservation and sustainable use of biodiversity, long-term human well-being and sustainable development [36]. That assessment provided a robust evidence base to test progress towards the Aichi Biodiversity Targets, helped to shape the Kunming-Montreal Global Biodiversity Framework, and to inform implementation of the Sustainable Development Goals and the Paris Agreement on Climate Change.

## 3. Impact of the institutional setting on the role of conservation scientists in policy

### 3.1. Academic research and higher education institutions

Many scientists in academia would resemble a *Science Arbiter* in Pielke's model, given that freedom of speech and action of its students and academics are fundamental to effective higher education and creative research and the role of higher education institutions (HEIs) to test and extend current understanding. Many academics will also act regularly as *Honest Brokers*, particularly when fulfilling government advisory roles in scientific assessment of policy. An increasing emphasis on the impact of a scientist's research for society more broadly in grant and fellowship evaluation processes and promotion applications is encouraging a more applied perspective, although applied impact may often be less valued in research funding decisions and in reward structures in academia compared to other valuable criteria (such as blue sky thinking).

Growing recognition of the scale of the biodiversity and climate crises and their increasing impact on society is also driving academia and research institutions to operate in more sustainable ways, to advocate for the immediate practical value of their science, to support staff choosing to engage in activism, and advocate for more progressive practices and values than other sectors [2]. In some cases, science faculties now appoint policy managers whose job it is to facilitate science–policy exchange, for example, through evidence-based policy briefs and reports (see [37]). While not without risks and challenges, for example, that the science might be seen as lobbying, such initiatives provide a valuable bridge between academic science, society and policy, at a time when one is critically needed.

Consistent with these institutional trends, a small but growing number of high-profile academics also act vocally as *Issue Advocates* on both climate and nature [38–42] and there is a trend, perhaps in the UK in particular, for research institutes themselves to carry mission, or value-driven labels and statements: for example, the *Leverhulme Centre for Nature Recovery*, the *Cambridge Conservation Research Institute*, the *Reading Centre for Climate and Justice*, the *Leverhulme Centre for Anthropocene Biodiversity* and the *Centre for Climate Crime and Justice*.

These trends may reflect the latest shift in a long history of adjustments to what scientists think they should do, and what they are able to do at the science–policy boundary, as political and social contexts change, and different priorities emerge. As Oppenheimer *et al*. [5] pointed out, during the mid-twentieth century many prominent professional scientists as well as philosophers and theologians spoke out as sentinels and advisers, campaigning to raise awareness of threats as varied as ozone depletion, acid rain, nuclear armament and fascism. Rachel Carson [43], a US Fish and Wildlife Service biologist, is revered in the environmental movement for her efforts to bring the damaging impacts of agricultural pesticides to the world's attention in the 1960s.

### 3.2. Non-governmental organizations and the RSPB as a case study

Trends seen in academia increasingly close the conceptual gap between HEIs and the global community of eNGOs and may partly explain the rise of long-term partnerships and collaborations across these sectors, such as the UK's Cambridge Conservation Initiative (CCI) [44]. Nonetheless, eNGOs typically remain more mission-driven than HEIs, with their work focused on public campaigning and influencing policy, though mostly founded strongly in evidence-based principles. Where those eNGOs manage land or seas directly, scientific evidence is also used and commissioned to inform and improve conservation practice [45]. Funders such as the CCI's Endangered Landscapes and Seascapes Programme [46] now require robust monitoring and evaluation and testing of novel intervention as conditions of funding. Many eNGOs also use scientific evidence to inform advocacy and activism as they work with governments, businesses, and the public to develop and pursue policy solutions for specific conservation or other problems. Policy advocacy strategies may be either ‘inside-track’ (i.e. working directly with governments and their agencies, or businesses, in formal processes of consultation and debate on policy formation—‘inside the tent’) or ‘outside-track’ (i.e. using public campaigns, headline-grabbing actions and arresting communications to exert pressure on governments in order to further policy goals—‘outside the tent’; see box 2). Both approaches may often be used and may interact in complex ways.

The RSPB is a large eNGO, the UK member of a global partnership, Birdlife International, a family of over 115 national organizations focused on bird and nature conservation. It is a UK nature conservation charity supported by over a million members with a large professional staff and annual budget (> £100 million) managing a network of over 200 nature reserves, engaging in policy advocacy and practice with the aims of creating a world richer in nature for nature and people, and tackling the twin crises of nature and climate (box 3). Similar goals are shared by many eNGOs worldwide, often garnering large public support and sizeable memberships in support of their mandate. Many eNGOs also have some in-house scientific capacity and expertise. However, the RSPB is unusual in having a dedicated and large team of scientists (approx. 60 permanent staff) engaged in research according to the scientific needs of the organization [47,48].

Box 3. **The Royal Society for the Protection of Birds (RSPB).**

**Charitable purpose.** The RSPB exists to deliver its charitable objects for the benefit of the public:

1. To promote the conservation of biological diversity and the natural environment for the public benefit, in particular, but not exclusively by: a. conserving wild birds and other wildlife, and the environment on which they depend; b. protecting, restoring and re-creating habitats. And, in furtherance of that primary objective, to raise public understanding and awareness of, and to provide information on, such matters: 2. To advance education of the public in conservation of the natural environment.

**Mission.** The RSPB is dedicated to creating a world richer in nature. We use our expertise in birds and nature to provide evidence-based solutions to the nature and climate emergency, helping people live well in harmony with nature. We work with our partners to keep common species common, recover threatened species, protect and restore special places and inspire and enable everyone to act for nature. We operate in the four countries of the UK, the Crown Dependencies and Overseas Territories. We also work globally, wherever our shared nature goes, or the need exists.

**Science.** The role of the science team at RSPB is to identify, provide and interpret the scientific evidence needed to help the charity, and the global partnership, Birdlife International, of which it is a part, to protect and conserve nature. Its science is designed to deliver solutions in a cycle that moves from identifying problems, through knowing causes, trialling solutions, and knowing that actions work [47,48]. More broadly its aim is to assist other societal actors to make well-informed decisions on nature conservation and the environment. The science team works closely with in-house policy and advocacy professionals and conservation practitioners, as well as with many external partners, from land managers and fishermen, to governments and Multilateral Environmental Agreements and fora.

**Organizational history.** The RSPB was formed to protest against the trade in bird plumes, a fashion responsible for the destruction of many thousands of egrets, birds of paradise and other species, whose plumes had become fashionable in the late Victorian era. In this sense, the RSPB was born of activism. The organization started life as the Society for the Protection of Birds founded by Emily Williamson in Manchester in 1889. The fledgling society was so successful that, having merged with other groups with similar intent, it was granted its Royal Charter in 1904. In 1921, the Importation of Plumage (Prohibition) Act was passed, forbidding plumage from being imported to Britain.

RSPB scientists resemble *Honest Brokers* in that they tackle scientific questions that are asked to inform conservation policy and practice. The criteria used to judge new research will overlap, but likely be different from those used to evaluate publicly funded research. However, like academic scientists, RSPB staff operate with rigour and objectivity following scientific practice, and as members of the wider scientific community, by engaging in peer-review, presentation of findings at scientific conferences, supervision of PhDs, and representation on scientific bodies and editorial boards. Some hold honorary professorships or lecturer roles within UK universities. However, they also engage with policy advocates (i.e. professional *Issue Advocates*), conservation practitioners and other decision-makers within and outside the organization, and so engage directly in the development of conservation policy and practice. To be credible and effective, RSPB scientists must therefore maintain high scientific standards (e.g. through peer-reviewed publication of their work and sharing their findings at national and international meetings), be able to review evidence impartially, and set out options for policy and decision makers and conservation practitioners. However, their dual role means they may not stand aloof from the discussions of policy and practice alternatives. Indeed, their role is sometimes as critical friends to convey ‘inconvenient truths’ in situations where new evidence challenges pre-existing practice, policy or values. At other times, they may be called upon to act, or find themselves, in an *Issue Advocate* role as representatives of their mission-driven organization. Here there is a fine line to tread and caution is needed, given blurring the lines between scientific and advocacy roles carries reputational risk to the organization and the individual scientist [5]. This illustrates that when working in a real-world discipline, like biodiversity science, scientists need to be able to operate as both effective scientific communicators and advocates, while maintaining their scientific credibility. This is a serious challenge to do well, requiring a high level of awareness of the roles being played and how these vary across contexts both by the scientists themselves, and by their colleagues whose conservation work they are informing with evidence.

Developing such skills and understanding is a challenge for all parties, but the need for this agility and awareness is especially acute when scientists are embedded within organizations delivering conservation practice and policy advocacy, as is the case in the RSPB. However, the benefits of such co-location can be great, as the efficiency with which evidence is translated and actioned can often be greater than in institutional settings where the ‘production’ and ‘consumption’ of science are separated. In this way, the RSPB's model of blended science, advocacy and practice has proven effective in delivering solutions to conservation issues [48] that would be much more difficult to achieve, or would take longer to achieve, otherwise. Examples, focusing on species and site conservation, include the design of agri-environment schemes to help farmland birds [49], the conservation of Asian vultures [50], recovery of Eurasian bittern (*Botaurus stellaris*) and cirl bunting (*Emberiza cirlus*) populations [51,52], legal protection of mountain hare (*Lepus timidus*) [53]*,* legal control of heather burning on protected sites [54]*,* protected area designation [55,56] and assessing the state of nature [57].

## 4. Challenges and risks at the interface of science and policy

**Figure 1.**
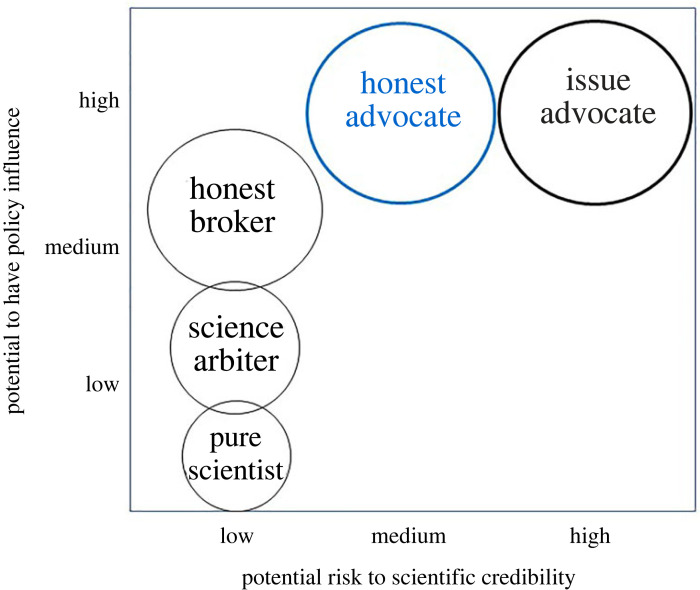
Five idealized roles of a scientist building upon Pielke's heuristic model but adding the ‘Honest Advocate’ and showing how the potential to have policy influence either directly or indirectly in decision making is related to the potential risk to scientific credibility and trust in science.

Pearson [24] eloquently describes the challenges of communicating science to different audiences, the dangers of exaggeration beyond published findings, and the pitfalls when a scientist acts as an advocate. The biggest danger is that societal trust in science could be undermined when scientific and advocacy roles become conflated (figure 1). That said, the risk to scientific credibility might be equally high if scientists failed to engage actively in advocacy too, especially when they judge threats to be extreme. Pearson uses the example of projected climate change impacts on biodiversity twenty years ago [58]*,* and hyperbole in the way a key paper was presented by the media. On the one hand, this paper put an invaluable spotlight on an emerging and underestimated societal threat, while on the other, Pearson noted the risk of ‘crying wolf’ [59] that could undermine the science and message that urgent action may be needed. At the same time, mass communication of science is extremely challenging and worthy, caveat-laden results are unlikely to be published in high-profile journals, or to attract media attention. It is also the case that society may have to tolerate a higher level of uncertainty about risk when recognizing urgent threats if we are to act sufficiently quickly to make a difference. The rapid testing and roll-out of COVID-19 vaccines across the world during the pandemic is an instructive example. It is also interesting that the burden of proof is always on those demanding change, even when existing forms of environmental economics drive damaging change, even if environmental catastrophe is not (yet) certain.

Pearson's essay highlights the pitfalls in science advocacy when it reflects personal values and preferences. However, when advocacy is evidence-based then that criticism does not apply, even if the scientist's motivation to work in a field *does* (as it often surely must) reflect their values. A scientist can, when presented with a specific policy goal, advise one intervention over another based on the best scientific evidence, independently of those personal views. Note also that an appropriate level of understanding of science and the scientific process among the policy makers would assist in this situation. Even-handedness should not be the sole responsibility of the scientist.

In conclusion, Pearson argues that scientific advisers must first stick to the science, remaining non-partisan, avoiding exaggeration, and setting out caveats and uncertainties; as the *Honest Broker* would. Secondly, scientists should also engage with issues of societal importance as citizens and have a special part and responsibility to play in that role, given the authoritative contribution they can make. This chimes with Oppenheimer *et al*.'s [5] view that while patrolling the science–policy border is important, scientists should not be reticent about intervening in debates about policy and practice where their expertise is relevant to the problem at hand. Furthermore, scientists have the *right* to intervene as other citizens do. Gardener *et al*. [2] go further in suggesting that the traditional academic roles of research, teaching and policy engagement are not sufficient to drive transformative change at a time of global crises, arguing for structural changes in academia to embrace and reward both advocacy and activism. This makes sense when current best available data [60] suggest that dramatic change in how economies value nature must occur in the next 5–10 years for there to be any latitude for meaningful action in the decades to come.

In this context, a key challenge is that the opportunity to be involved in *Honest Broker* or *Issue Advocate* roles is most frequently held by scientists who already occupy privileged, secure positions, introducing structural bias in which voices are heard. Scientists who choose to engage in political debate and activism may typically be in tenured positions where academic freedom and organizational trust allows them to operate in a safe space, while some may be retired and have greater security to act in a personal capacity, with additional traction provided by their scientific track record and reputation. However, for an early career scientist without such security, direct engagement in advocacy or activism poses a real dilemma, since criteria used to justify career progression are less likely to value such roles, and if such roles lead (as they currently do) to questions about objectivity, then this may bring career disadvantage. In addition, if any activism was to lead to a criminal conviction that would also count against their future employment prospects.

## 5. Conclusion and a way forward

We live at a time of extreme environmental change that, apart from looking likely to remove many of the other species and ecological communities from the planet, poses an existential risk to future human prosperity, quality of life and social justice. Collective failure to act decisively this decade looks likely to condemn our children, as well as all but the wealthiest, to a greatly diminished and less productive future on this planet. Increasing frustration across society with a lack of sufficient (and often promised) progress by governments and business in tackling those issues has led to a rise in activism and direct action, in the form of public protests across the world, focused on environmental challenges. Whether such activity promotes or deters positive action to address the climate and biodiversity crises is itself contested [2]*.* However, some scientists, in either professional or personal capacities, engage in such activism, demonstrating their strength of conviction, and often argue that advocating in every way possible for a liveable future is a rational rather than political act. A conviction that environmental destruction is an existential threat seems common among biologists, and so presents a dilemma in how best to integrate a scientific career, where one's professionalism revolves around evidence and impartiality, with one's moral values and responsibilities, and a desire to act to do good. We note that this tension might be particularly acute for early career scientists and structurally disadvantaged groups who have less freedom to act and engage with policy.

We offer no simple solution to squaring this circle, other than to recognize that a scientist may need to work across several of Pielke's idealized roles to meet the needs of a society facing acute change, as well as their responsibilities to it, and that by recognizing those roles and their characteristics, we might achieve more impact as scientists and citizens. We note that such duality is to be expected, given that scientists do not choose their research fields by accident; instead, they study parts of nature that give them joy. The emotions and anxiety they feel as they document the loss of nature should therefore come as no surprise. However, we do not recommend scientists always act as out-and-out *Issue Advocates*, because that role is often best played by others and risks of undermining confidence in science (figure 1). At the same time, we question whether it is enough for scientists to act solely as *Honest Brokers* in a society under threat through existing policies and economies, and stand away from advocacy or campaigning completely, given the urgent need for meaningful action. The *Honest Broker* simply sets out options to allow freedom of choice for decision makers. It is claimed by some that scientists cannot advocate without exposing personal values and preferences, although we note that these personal values and preferences are rarely seen as a disadvantage for those deciding health policy. Instead, we argue that scientists can and should advocate for one course of action over another if this is based upon their expert interpretation of the scientific evidence. At this stage, objectivity, professionalism and transparency are key. However, this should not prevent scientists also advocating for one of those goals from a personal and emotional belief, as is their responsibility as citizens and humans provided their motivation is made plain. The challenge is for scientists' roles as citizens (and their engagement in protest as well as policy) to be seen as legitimate rather than suspicious by those outside science.

We suggest that a scientific role emerging from Pielke's work is one where a scientist maintains and upholds scientific principles, is transparent and open, but requires certain conditions to be met to maintain their legitimacy. This role is perhaps best described as the *Honest Advocate* [61]*.* From the RSPB's perspective, a good example is the work to assess the efficacy of nature protection measures in the European Union (EU) [62–65]*.* This research showed how two nature directives, the Birds Directive of the EU [66] and the Habitats Directive of the EU [67] (92/43/EEC), delivered very effective protection of the special birds and sites they target, and so has been used with other research to defend the directives from weakening; helping to shape ambitions within a newly devised EU Nature Restoration Law [68]. In this example, knowledge was honestly compiled and presented in a rigorous scientific fashion and was presented to improve the quality of advocacy for specific outcomes, narrowing and weighting choices based on transparent criteria, rather than expanding them [61]*.* Importantly, the *Honest Advocate* (role 5) also retains considerable agency to contribute to decision making in a purposeful and well-informed manner, doing societal good, but without damaging the good reputation of science (figure 1). We encourage each scientist to reflect on their place in society and to consider when it might be appropriate to act as an *Honest Advocate*, alongside other valuable roles given the urgent environmental challenges of the twenty-first century.

## Data Availability

This article has no additional data.
